# Concordance between medical records and interview data in correctional facilities

**DOI:** 10.1186/1471-2288-14-50

**Published:** 2014-04-09

**Authors:** Jennifer R Bai, Dhritiman V Mukherjee, Montina Befus, Zoltan Apa, Franklin D Lowy, Elaine L Larson

**Affiliations:** 1 School of Nursing, Columbia University, New York, NY, USA; 2 Department of Epidemiology, Mailman School of Public Health, Columbia University, New York, NY, USA; 3 Department of Medicine, Division of Infectious Diseases, College of Physicians and Surgeons, Columbia University, New York, NY, USA

**Keywords:** Medical records, Interviews, Questionnaires, Self-reports, Concordance, Reliability, Agreement, Kappa statistics

## Abstract

**Background:**

Self- administered questionnaires or interviews and medical records are often used as sources of research data; thus it is essential to evaluate their concordance and reliability. The aim of this paper was to assess the concordance between medical and behavioral data obtained from medical records and interview questionnaires in two correctional facilities.

**Methods:**

Medical record and interview data were compared for 679 inmates from one male and one female maximum security prison between April 2010 and February 2013. Gender non-stratified and gender-stratified analyses were conducted in SPSS to calculate the prevalence and kappa coefficient scores (κ) for medical (e.g., HIV, diabetes, hypertension) and behavioral (e.g., smoking, drug use, tattoos) conditions. Sensitivity/specificity between medical records and interview were calculated in the gender non-stratified data.

**Results:**

In the gender non-stratified analysis, κ score for HIV, hepatitis C, diabetes, asthma, and history of tattoos had strong or good concordance (0.66-0.89). Hypertension, renal/kidney disease, cigarette smoking, antibiotic use in the last 6 months, and cocaine use ever were moderately correlated (0.49-0.57). Both history of any illicit drug use ever (0.36) and marijuana use ever (0.23) had poor concordance. Females had higher κ scores and prevalence rates than males overall. Medical conditions were reported more frequently in medical records and behavioral conditions had higher prevalence in interviews. Sensitivity for medical conditions in the combined facility data ranged from 50.0% to 86.0% and 48.2% to 85.3% for behavioral conditions whereas specificity ranged from 95.9% to 99.5% for medical conditions and 75.9% to 92.8% for behavioral conditions.

**Conclusion:**

Levels of agreement between medical records and self-reports varied by type of factor. Medical conditions were more frequently reported by chart review and behavioral factors more frequently by self-report. Data source used may need to be chosen carefully depending upon the type of information sought.

## Background

Medical records, interviews and self-administered questionnaires are frequently used sources of research data, thus many studies have assessed the concordance and reliability between these data collection methods. Although some researchers have considered medical records as the “gold standard” and the preferred data source over questionnaires, evidence indicates that neither source is completely accurate and that combining sources may result in a more reliable and complete data assessment [1-5]. Any method of data collection can introduce its own sources of measurement error. Questionnaire data, either from self-report or interview, have limitations such as recall bias, misinterpretation of the questions and degree of willingness to report. On the other hand, medical record data may also be limited by illegibility; incomplete, inaccurate or missing documentation; and limited availability of data elements [2,6-8]. Furthermore, medical records are designed for clinical rather than research purposes. Thus, multiple challenges exist regarding what data to extract and how, especially when multiple investigators are involved if a standardized extraction protocol is not established [9,10].

While published population-based studies have examined the concordance between medical records and questionnaire data for chronic medical conditions and its related symptoms [3,4,11-13], we did not find literature within the past decade that summarized and compared the reliability and concordance between these two data sources in an incarcerated population. Because we were collecting data for a research study and drawing inferences from these data which could have an impact on clinical care and policies (see ‘Study population’ below), it was essential to determine the extent to which various data sources were complete, available, and consistent. Therefore, the aim of this study was to assess the concordance between medical records and interview questionnaires for medical and behavioral conditions obtained from two maximum security correctional facilities.

## Methods

### Study population

We used medical record and interview data collected from an ongoing study, “Risk Factors for Spread of *Staphylococcus aureus* in Prisons” (NIH, ROI AI82536), which was approved by the institutional review boards of New York State Department of Corrections and Community Supervision (DOCCS) and Columbia University Medical Center. This study is being conducted in two maximum security prisons: Bedford Hills Correctional Facility for Women which houses about 900 inmates at Bedford Hills, NY and Sing Sing Correctional Facility for Men with about 1800 inmates at Ossining, NY [14]. The method of recruitment of inmates was tailored to the processing regulations and safety policies of the two prisons and has been previously described [15]. Eligibility for participation included: (1) at least 16 years of age, (2) introduced into the general incarcerated population for at least six months, and (3) ability to speak and read English.

### Collection of data sources

After obtaining signed informed consent, a trained research assistant interviewed the inmate in a private room using a structured questionnaire which included questions regarding demographics, education level, daily activities and general hygiene. Participants were asked more specifically regarding personal medical history (e.g., “Do you have any pulmonary disease such as asthma?”), previous skin infections, antibiotic use in the last six months, current tobacco/cigarette smoking and history of drug use. Correctional officers escorted them to the room but were not present during the interview process. Medical records data were collected independently following the interviews for those who agreed to participate in the study.

### Data extraction

Medical record data were extracted from paper-based medical charts by two fulltime, training research assistants. All records were filed in order of the inmate’s identification number in the medical record office and were easily accessible by the research assistants. All inmate medical records consisted of a medical history problem list, handwritten notes from health care providers, psychiatric information, laboratory results, drug prescription charts, and physical examination reports that included drug use history and sexual orientation. For the concordance analysis, we extracted variables that were previously examined in the literature as well as other variables of interest that were available from both the medical records and interview questionnaires. Information regarding antibiotic use in the six months prior to the interview date regardless of their site of residency was found mainly in the handwritten notes and sometimes in the drug prescription charts, if present in the inmate’s file. From the list of medical history and the most recent physical examination reports, we were able to identify the following: chronic medical conditions such as asthma, diabetes, renal/kidney disease, hypertension (including those who reported high blood pressure), hepatitis C, and HIV, and behavioral conditions including current cigarette smoking, and history of tattoos, marijuana use, cocaine use, and any illicit drug use.

### Statistical analyses

Medical records and interview questionnaire data were analyzed in IBM SPSS Statistics Version 20 (SPSS, Inc., Chicago IL, USA). The prevalence of each variable was calculated from each source. The kappa (κ) coefficient was computed to assess the concordance between the medical record and interview for each variable of interest. The kappa value (0.00 to 1.00) was categorized as suggested by Landis and Koch, 1977 [16] as: poor (< 0.20); fair (0.21-0.40); moderate (0.41-0.60); good (0.61-0.80) and strong (0.81-1.00). Two separate analyses were conducted, gender non-stratified and gender stratified, to compare any differences in the kappa score and prevalence by gender. Because the results between the two analyses were similar, sensitivity and specificity were only calculated using the gender non-stratified data. Similar to Tisnado et al. [5], we also considered the data source with the highest prevalence (either medical records or interviews) as the ‘gold standard’ when calculating sensitivity and specificity.

## Results

### Population characteristics

In the two correctional facilities, 801 inmates participated in the parent study between April 2010 to February 2013 (participation rate = 82.7%). A total of 679 participants for whom that had all the variables of interest presented in both interview and medical record data were included in this analysis. As summarized in Table 1, more than half of participants were black non-Hispanic (53.5%), followed by white non-Hispanic (22.5%) and Hispanic (21.4%). There were more female (55.5%) than male (44.5%) participants, and ages < 25 years old (15.9%), 26–35 years old (30.9%), 36–50 (43.2%), > 51 years old (10%), mean: 37 years.

**Table 1:** Demographics, gender and age of inmate participants

**Characteristics**	**Study population N = 679 (%)**
**Sex**	
Male	302 (44.5%)
Female	377 (55.5%)
**Age, years (mean age = 37)**	
< 25	108 (15.9%)
26-35	210 (30.9%)
36-50	293 (43.2%)
> 51	68 (10.0%)
**Race/Ethnicity**	
White non-Hispanic	153 (22.5%)
Black non-Hispanic	363 (53.5%)
Hispanic	145 (21.4%)
Others	18 (2.65%)

### Prevalence of medical condition variables

In the gender combined analysis, prevalence rates of all variables in the medical condition category were higher in the medical records when compared with the interview questionnaires. Asthma (39.6%) was the most prevalent condition and renal/kidney disease (2.5%) was the least prevalent condition among the inmates. Females had a higher prevalence of both medical and behavioral conditions in both data sources when compared to males, with asthma still as the most prevalent condition (females- 47.2% vs. males- 30.1%) and renal/kidney disease as the least prevalent condition (females- 3.2% vs. males- 1.7%). For females only, all medical conditions were reported more frequently in medical records when compared with the interviews, except for diabetes (9.1% vs. 9.3%) and renal/kidney disease (3.2% vs. 3.7%) (Table 2).

**Table 2:** Prevalence of medical and behavioral conditions are reported in medical records and interviews by male and female participants

**Medical Conditions**	**Males**		**Females**		**Overall Prevalence**	
	**% (N = 302)**		**% (N = 377)**		**% (N = 679)**	
	**Medical record**	**Interview**	**Medical record**	**Interview**	**Medical record**	**Interview**
Asthma	30.1	21.5	47.2	39.3	39.6	31.4
Hypertension	16.6	10.3	22.5	17.2	19.9	14.1
Hepatitis C	10.6	5.3	18.3	12.2	14.9	9.1
Diabetes*	4.9	3.9	9.1	9.3	7.2	6.9
HIV	2.3	1.9	9.5	9.0	6.3	5.9
Renal/kidney disease	1.7	1.3	3.2	3.7	2.5	2.7
**Behavioral conditions**	**Males**		**Females**		**Overall Prevalence**	
	**% (N = 302)**		**% (N = 377)**		**% (N = 679)**	
	**Medical record**	**Interview**	**Medical record**	**Interview**	**Medical record**	**Interview**
Cigarette smoking*	60.4	64.4	74.0	82.0	67.9	74.2
All illicit drugs use ever*	50.8	81.9	78.0	85.4	66.1	83.8
Tattoos	48.7	61.6	57.0	57.0	53.3	59.1
Marijuana use ever*	37.8	78.6	41.3	75.2	39.9	76.6
Cocaine use ever*	19.4	24.1	48.4	55.0	35.7	41.3
Antibiotic usage in last 6 months*	17.9	23.9	42.0	43.1	31.5	34.6

*Denominator excludes missing data.

### Prevalence of behavioral condition variables

For the gender combined data, all behavioral condition variables were more likely to be reported in the interview questionnaires than in the medical records including any illicit drug use ever (83.8% vs. 66.1%, respectively), marijuana use ever (76.6% vs. 39.9%, respectively), and antibiotic use in the last six months (31.5% and 34.6%, respectively). After stratifying by gender, behavioral conditions were still more likely to be reported in the questionnaires except for history of tattoos in the female, which had the same reported prevalence rates in both questionnaire and medical record (Table 2).

### Concordance between medical records and interview questionnaires

The kappa coefficients for the combined facilities and gender stratified analyses ranged from 0.19 to 0.91, with all scores statistically significant (P-value < 0.01). In the gender combined data, HIV (κ = 0.89) and diabetes (κ = 0.82) had the highest kappa scores between medical records and questionnaires, followed by asthma (κ = 0.78), hepatitis C (κ = 0.66), and history of tattoos (κ = 0.76) whereas marijuana use ever (κ = 0.23) and any illicit drug use ever (κ = 0.36) had the lowest kappa scores. In general, variables in the medical condition category had better kappa statistics (range κ = 0.50-0.89) compared to variables in behavioral condition category (range κ = 0.23-0.76).

Overall, females had higher kappa scores than males in both medical and behavioral condition categories. The only kappa score lower among females than males was cigarette smoking, although the differences were small (κ = 0.51 vs. 0.55, respectively). Marijuana use ever (κ = 0.19) and any illicit drug use ever (κ = 0.26) had the lowest kappa scores in males, whereas the female population scored almost twice as high in both variables (Table 3).

**Table 3:** Comparison of agreement between medial records and inmate interviews: Kappa coefficients, sensitivity and specificity

**Medical condition**	κ coefficient**			**Medical record as gold standard (Overall)**	
	**Male**	**Female**	**Overall**	**Sensitivity (%)**	**Specificity (%)**
HIV	0.76	0.91	0.89	86.0	99.5
Diabetes*	0.81	0.82	0.82	81.6	98.9
Asthma	0.73	0.81	0.78	76.9	98.5
Hepatitis C	0.55	0.71	0.66	56.4	99.1
Hypertension	0.51	0.60	0.57	54.8	95.9
Renal/kidney disease	0.44	0.52	0.50	50.0	98.8
**Behavioral conditions**	κ coefficient**			**Interview questionnaires as gold standard (Overall)**	
	**Male**	**Female**	**Overall**	**Sensitivity (%)**	**Specificity (%)**
Tattoos	0.65	0.87	0.76	85.3	92.8
Cigarette smoking*	0.55	0.51	0.55	83.0	75.9
Antibiotic usage in last 6 months*	0.45	0.51	0.51	64.1	86.0
Cocaine use ever*	0.43	0.43	0.49	63.7	84.1
All illicit drugs use ever *	0.21	0.54	0.36	74.6	77.9
Marijuana use ever*	0.19	0.26	0.23	48.2	87.3

*Denominator excludes missing data.

**P-values for all variable are significant (P < 0.01).

### Sensitivity and specificity

Using medical records as the gold standard in the medical condition category, sensitivity ranged from 50.0% to 86.0% and specificity from 95.9% to 99.5%. Sensitivity was < 60% for hepatitis C, hypertension, and renal/kidney disease. HIV (86.0%) and diabetes (81.6%) had the highest sensitivities. Specificity was high for all the variables, ranging from 95.9% to 99.5%.

The questionnaire was used as gold standard for the behavioral condition category. Sensitivities for behavioral conditions ranged from 48.2% to 85.3% and for specificity, 75.9% to 92.8% Tattoos had the highest sensitivity (85.3%) and marijuana use ever was the lowest (48.2%). Specificity was lower and less consistent than that of the medical condition category, ranging from 75.9% for cigarette smoking to 92.8% for tattoos (Table 3).

## Discussion

We evaluated the concordance between medical records and interview questionnaires for medical and behavioral conditions in two incarcerated populations in New York State. Similar to the findings of Schofield et al. [17], we found that inmates were generally reliable respondents for health-focused surveys. Overall, our findings were consistent with previously published studies conducted in either a community or clinical settings; the prevalence of chronic medical conditions except for renal/kidney disease was higher in the medical records when compared to the questionnaires, which could be due to under-reporting in the interviews, as has been previously reported [13,17-20]. Similarly, behavioral conditions are likely to have been under-reported in the medical records, especially for variables such as drugs and antibiotics. In contrast to a literature summary by Garber et al. [21], we found that interviews had just as good concordance as self-administered questionnaires when compared with medical records.

Like Okura, et al. [3], which was published almost a decade ago, and Malik et al. [19], a more recent study, we also found strong concordance between medical records and questionnaire responses for HIV and diabetes, which could indicate that most participants were aware of their diagnoses and willing to disclose that information [3,12,13,19,20].

Consistent with findings of Iversen, et al., Leikauf et al., and Tisnado, et al., we also found good concordance for reporting of asthma [5,13,18]. Hepatitis C, on the other hand, had a lower concordance level and was more likely to be reported by medical record, suggesting that participants were either unaware of their status or unwilling to report to the investigators.

In contrast to medical conditions, all behavioral conditions were reported more frequently in the interview questionnaires than in the medical records. Reports on history of any illicit drug use and marijuana use had the lowest kappa scores and the greatest difference between the two sources, perhaps because inmates are less likely to report drug use to health care providers during the physical examination than to the interviewers in fear of reprehension. Our kappa scores for current cigarette smoking and cocaine use were slightly lower than those reported in a previous study [19]. Since certain medical information was not up to date, the most recent reports of current cigarette smoking might not be representative of the inmate’s current smoking habits. A history of tattoos, on the other hand, had the best concordance and highest sensitivity/specificity in behavioral conditions. This could be explained by the fact that tattoos are noticeable and legal, thus inmates may not be wary of reporting them. Overall, interview questionnaires may be a better source of data for behavioral conditions than the medical record.

After gender stratification, no significant differences were found as compared to the gender non-stratified analysis; however, females did report much higher prevalence and concordance levels than males for all variables, also consistent with previous research [20,22]. Since both facilities have similar medical care accessible to inmates, this difference could be due to actual higher prevalence of conditions in females, the fact that females may be more aware and health conscience than males, therefore more willing to share information, or that females tend to frequent medical unit more often than males. Further studies on gender differences should be conducted to clarify these distinctions.

This study had limitations and bias that could have affected our findings. The incarcerated population may not be generalizable to other populations. As previously reported, medical records are often incomplete, missing information, or not up to date [2,7,17,18]. Specifically in this study, the medical records were handwritten, not electronic records like in the population-based studies, thus it was difficult to retrieve the necessary or, at time, accurate information. We did not record the length of time required to extract data from the medical records, but it varied considerably, depending upon the handwriting in the notes. Clearly, data extraction would be greatly facilitated in electronic medical records.

Because two research assistants were conducting the interviews and extracting from the medical charts, there could be variations in data collection processes and interpretation. However, this did not have any significant effect on the data analysis or results, since the medical record form was straightforward and both research assistants were trained to follow a standardized extraction protocol. Furthermore, studies have shown high kappa scores and percent agreement of intra- rater and inter-rater reliability for medical record extraction [10,23,24]. Most importantly, for many of the variables we examined, particularly the behavioral factors such as sexual practices, it was not possible to assess validity because there was no confirmatory ‘gold standard’. Nevertheless, our findings should provide some guidance as to when the medical record or self-report might be the most reliable data source.

## Conclusion

Medical records and self-reports are often data sources used in research, thus it is essential for investigators to analyze the concordance between the two for any variables of interest. While our findings were similar to what has been previously reported for chronic medical conditions regardless of the dissimilarity in study population, the level of concordance between the two sources varied greatly depending on the variable. Hence, investigators should choose data sources and construct questionnaire forms carefully depending on the population and variables of interest.

## Abbreviations

DOCCS: New York State Department of Corrections and Community Supervision; NIH: US National Institutes of Health.

## Competing interests

The authors declare that they have no competing interests.

## Authors’ contributions

RB was involved in conceptualization, literature search, writing and data interpretation of the study. DVM and MB contributed to editing the manuscript and data analysis. ZA helped with data collection. All authors read and approved the final manuscript.

## Pre-publication history

The pre-publication history for this paper can be accessed here:

http://www.biomedcentral.com/1471-2288/14/50/prepub
